# Why do democracies respond differently to COVID-19? A comparison of the United States and South Korea

**DOI:** 10.3389/fpubh.2023.1285552

**Published:** 2024-01-15

**Authors:** Yexin Mao

**Affiliations:** School of Public Administration, Hangzhou Normal University, Hangzhou, China

**Keywords:** democracies, crisis leadership, political and social solidarity, crisis management, COVID-19

## Abstract

**Background:**

COVID-19 has posed severe challenges to crisis management in democracies. Different democracies respond to the crisis differently. This article proposes an analytical framework to explain why democracies respond differently to the public health crisis and how different contextual factors affect crisis response in democracies.

**Methods:**

By comparing COVID-19 responses in the US and South Korea, this article conducts a comparative case study with a most similar system design. The two countries have been selected as cases because they are both developed democracies with a robust healthcare system. However, different contextual factors in the two countries have created different crisis responses by shaping different crisis leadership and political and social solidarity. This study collected data from different sources, including government documents, official websites, leaders’ speeches, research reports, academic articles and news media. We tried to enhance the reliability of the data by comparing different data sources.

**Results:**

We found that individual, institutional and cultural dimensions of contextual factors can influence different crisis responses of democratic countries by shaping crisis leadership and political and social solidarity. On the individual and institutional dimensions, leadership style and governance structure shape crisis leadership (sense making, decision making and coordinating, and meaning making), which in turn influences crisis management. On the cultural dimension, political and social solidarity measured by political polarization and social cooperation are shaped by cultural and social norms.

**Conclusion:**

Our findings indicate that democracies require strong crisis leadership and a high degree of political and social solidarity to tackle public health crises. A centralized and coordinated system, as well as a political elite leadership style shaped by rich crisis response experience, expertise and high sensitivity to crises are conducive to crisis management. Fostering a cultural and social norm that facilitates state–society collaboration can promote crisis management. These findings provide valuable insights for decision-makers to effectively respond to future pandemics.

## Introduction

Studies have shown that, compared with authoritarian regimes, democracies can rely on independent and diverse information sources such as civil society and free media for timely crisis detection through information collection and disclosure (1, 2). However, COVID-19 poses unprecedented challenges to democratic governance (3, 4). Different democracies respond to the crisis differently (5–7). In some democracies, their information advantage has not prevented epidemic outbreaks. For example, the Trump administration failed to promptly take effective measures after the outbreak, which led to the large-scale spread of the epidemic in the United States (8). In contrast, other democracies such as New Zealand and South Korea tackled the crisis effectively through quickly adopting measures after the outbreak (6). Why do democracies respond differently to public health crises? How do different contextual factors affect crisis response in democracies? Answering these questions is crucial to deepen our understanding of democratic resilience and public health crisis management in democracies (9).

Scholars have found some factors (e.g., preparedness, political support, institutional infrastructure, policy instruments, state capacity and ability to learn from previous crises) to explain different crisis responses between different countries such as democracies and authoritarian states (2, 10–15). Some studies further analyze why democratic countries respond differently to crises. For example, some scholars found that the strong protection of fundamental democratic principles and individual freedoms would make the government more reluctant to take strict and compulsory measures to limit individual freedoms and strengthen social control, which is not conducive to preventing the spread of the epidemic (5). Different intergovernmental relationships can shape the crisis response of democratic countries (16). For example, the federal system is more conducive to solving the challenges of diverse regions, and the unitary governance system is conducive to reducing intergovernmental conflicts (17). Different federal systems also affect different crisis management in democratic countries (7). A more centralized democracy with an independent healthcare sector can respond to crises more quickly (13).

In countries with a higher degree of democracy, the government will be more sensitive to the impact of other countries and more likely to learn from other countries’ effective measures to cope with crises (18). Countries with higher levels of democracy and higher citizens’ trust in government are more likely to respond effectively to public health crises (19). Differences in cognition of experts and political leaders, as well as differences in the urgency of crises also affect crisis response in democracies (20, 21). A stable decision-making structure that can institutionalize expertise into crisis response will be more conducive to efficient crisis management (22). The differences in the roles of the administrative and legislative departments in democracies will also shape different crisis responses (23). In addition, different types of democracies in terms of welfare state, such as liberal states, the conservative/corporatist welfare democracies, social welfare countries, will also take different welfare responses to crises (24). Although there are differences in crisis management measures in different democratic countries, effective democratic institutions and strong state capacity are the key to the democracies’ crisis response (25).

The existing research has discussed a few factors affecting the crisis response of democratic countries. However, these studies lack a comprehensive and systematic analytical framework to explore the specific mechanism of different contextual factors affecting crisis management in democracies. This article can make contributions to the literature on public health crisis management by filling this gap. By synthesizing and expanding the existing research, this article proposes a context-contingent integrative framework to analyze the specific mechanism of contextual factors influencing crisis management, and explains how different contextual factors affect the crisis response of democratic countries.

Specifically, contextual factors include individual, institutional and cultural dimensions. At the individual level, the leadership style of political actors can influence crisis management by shaping crisis leadership. At the institutional level, governance structure is an important factor in shaping crisis leadership and thus influencing crisis response. At the cultural level, cultural and social norms can influence crisis response by shaping political and social solidarity. These three dimensions of contextual factors can influence different crisis responses of democratic countries by shaping crisis leadership and political and social solidarity.

Crisis leadership includes sense making, decision making and coordinating, and meaning making. These three capacities can help governments promptly detect crises through information collection and analysis, quickly make decisions, coordinate different agencies and strengthen crisis communication with citizens, thereby enhancing crisis leadership. Political and social solidarity are measured by political polarization and social cooperation, respectively. A low degree of political polarization and a high degree of social cooperation can promote effective policy implementation in crisis management.

Furthermore, the existing research shows that crisis leadership and political and social solidarity play an important role in crisis management, but these studies have insufficient research on what factors affect crisis leadership and political and social solidarity. Our analytical framework demonstrates that governance structure and leadership style can influence crisis response by shaping crisis leadership. A centralized and coordinated system is more conducive to overcoming the bureaucratic fragmentation problem and promoting effective intergovernmental coordination and cooperation to tackle crises. Political elites’ leadership styles are more conducive to crisis management if they are informed by rich crisis response experience, expertise and high sensitivity to crises. Additionally, cultural and social norms can influence crisis response by shaping political and social solidarity. A strong cultural tradition of citizen-government collaboration is more conducive to reducing political polarization and social conflicts, thereby enhancing public trust in the government and the government’s capacity to manage crises. These findings can further deepen our understanding of public health crisis management in democratic countries.

This study selects the US and South Korea as cases in point. As mature democracies, both countries have robust public health systems, but they differ greatly in terms of containing COVID-19, so comparing the two countries can provide us with valid evidence to demonstrate the analytical framework and address our research questions. In order to effectively compare the crisis responses of different democracies, this article mainly focuses on the role of the central government (the Trump administration and Moon Jae-in administration) in the early stages of the outbreak in the United States and South Korea, and the interaction between the central government and subnational governments, for two reasons.

First, subnational governments in democratic countries have relatively strong autonomy, and local officials are elected by voters. Therefore, subnational governments are likely to respond to the crisis differently. For instance, under the federal system of the United States, both the federal government and state governments play important roles in public health governance (26). Different states can take different measures to deal with the public health crisis (27). However, the COVID-19 pandemic is a major national or even global public health crisis, which cannot be dealt with by subnational governments alone, and the central government plays an extremely important role in crisis response (28–30). Monitoring and early warning of global public health crises has become an important responsibility of the central government (28).

Second, because the crisis has great uncertainty and suddenness in the early stage of the epidemic, it can fully test the capacity of different democratic countries’ central governments to tackle the crisis. Moreover, at this stage, the central governments’ responses to the crisis in democratic countries are quite different, which can provide valid evidence for verifying our analytical framework. With the development of the epidemic, although democracies have encountered several different waves of the epidemic, due to the mutual learning of crisis management experience among democratic countries and the deepening understanding of the crisis by governments, the differences in crisis response among democracies are gradually decreasing.

This study first introduces the analytical framework, case selection and data collection, then analyses the two cases based on the framework. Lastly, it summarizes the major findings and discusses implications.

## Analytical framework

Crisis leadership and political and social solidarity play a crucial role in crisis response. First, the government requires strong crisis leadership to tackle complex crises (31, 32). Governments with strong crisis leadership can detect crises in a timely manner, conduct scientific analysis, make quick decisions, and strengthen crisis communication with society, so as to effectively manage crises (31). Moreover, crisis leadership also requires leaders’ ability to effectively interact and collaborate with other actors, mobilize them to actively participate in crisis response, form productive interactions and partnerships, and gather resources from different parties to effectively respond to crises (33–35). Crisis leadership is also reflected in the leaders’ ability to dynamically and flexibly adjust policies according to changes in the external environment to effectively respond to crises (36).

Second, political and social solidarity are another important factor affecting crisis management. For example, political solidarity (e.g., similarity and homogeneity between political actors) can alleviate the collective action dilemma, facilitate interlocal cooperation and enhance mutual commitment, thereby allowing effective crisis responses (37). Social solidarity is also conducive to overcoming crises through citizen-government collaboration and state–society synergy (38, 39).

However, which factors influence crisis leadership, as well as the political and social solidarity that advance effective crisis response, are underexplored. Studies have shown that contextual factors have a profound impact on government policymaking and crisis response (40, 41). For example, Petridou and Zahariadis (42) argued that institutional, administrative and political factors are three important contextual factors affecting crisis response. Others emphasized that institutional and cultural factors are key to shaping crisis management (14, 43). However, these studies ignore the impact of leaders’ personal contextual factor on crisis management.

**Figure 1:**
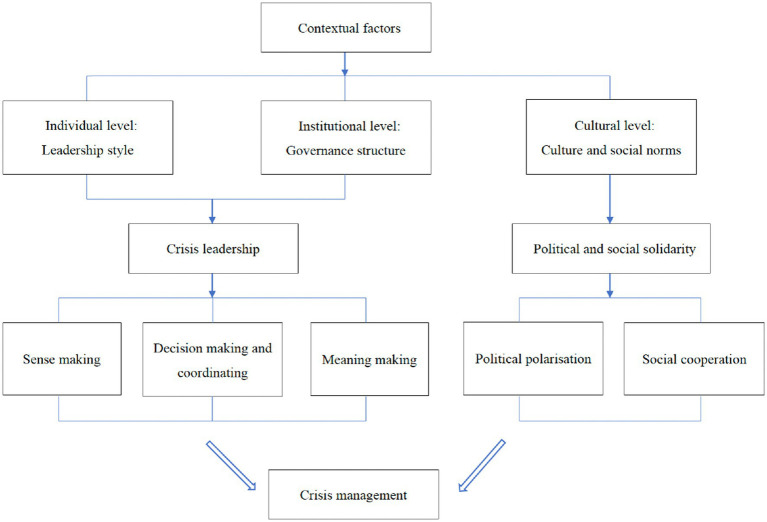
Analytical framework.

Drawing on these studies and the theories of crisis leadership and political and social solidarity, this article proposes a context-contingent integrative framework to analyze the specific mechanism of contextual factors influencing crisis management, and explains how different contextual factors affect the crisis response of democratic countries (see Figure 1). In this framework, crisis leadership and political and social solidarity are shaped by contextual factors including individual, institutional and cultural dimensions. In addition to the institutional and cultural dimensions discussed in existing research, we also added the leaders’ personal contextual dimension to our framework to examine how contextual factors affect crisis management. Specifically, in terms of individual and institutional dimensions, leadership style and governance structure shape crisis leadership, which in turn influences crisis management through sense making, decision making and coordinating, and meaning making. Moreover, political and social solidarity are measured by political polarization and social cooperation, respectively, and are shaped by cultural and social norms in terms of cultural dimensions.

### Crisis leadership

This study draws on the crisis leadership theory of Boin et al. (31), who proposed five tasks of government strategic crisis leadership: sense making; decision making and coordinating; meaning making; accounting; and learning. Due to the ongoing spread of COVID-19, this article focuses on sense making, decision making and coordinating, and meaning making because these are closely related to crisis detection and handling, while accounting and learning emphasize assessment of crisis management and lesson learning at the end of a crisis. Sense making, decision making and coordinating, and meaning making are closely related to governance capacity (e.g., prepardenss, coordination capacity, analytical capacity, regulatory capacity, delivering capacity) and governance legitimacy (e.g., social trust in the government) (44, 45).

Sense making refers to the government’s capacity to promptly detect the crisis through information collection and processing based on scientific expertise (31, 46). It is closely related to preparedness and analytical capacity, which emphasizes that at the early stage of the crisis, the government can strengthen information collection and processing, scientifically analyze risks according to the opinions of experts, quickly detect the crisis, issue crisis warning, and prepare for crisis response in advance (44, 46, 47). Effective information collection, analysis and sharing can help detect potential risks and improve public cognition of crises. It is important to establish a technology-driven integrated information management system to issue risk warnings and strengthen crisis analysis (48).

Decision making and coordinating refers to governments being able to make decisions quickly and to promote policy implementation through effective coordination (31). Decision making and coordinating are closely related to coordination capacity, regulatory capacity and delivery capacity. They mainly emphasize that when a crisis occurs, the government can make rapid and scientific decisions, effectively coordinate different levels of government, different departments and organizations to share information and resources to tackle the crisis (44). The government should also comprehensively consider the crisis risk, time and resource constraints and political feasibility to make anti-crisis decisions (49, 50). Furthermore, by strengthening the decision-making and inter-organizational coordination capabilities, the government can improve the social regulatory capacity and delivery capacity, enhance effective and flexible control of society, promote the implementation of the anti-crisis policies, and provide efficient public services for citizens, thus minimizing the negative impact of strict crisis response measures on economic and social development (45).

Meaning making refers to governments providing citizens with a convincing narrative to explain the occurrence of a crisis, the current situation and the anti-crisis measures being taken (31). It is very important for leaders to strengthen communication with citizens, provide them with reliable information and guidelines, and promote their cooperation with the government (51). Meaning-making is closely related to governance legitimacy. It mainly means that the government can strengthen crisis communication with the society to enhance the social trust in the government and improve the legitimacy of governance, so as to deal with the crisis more effectively. For example, by strengthening the openness of information, the government can shape public cognition of a crisis and enhance citizens’ evaluation of the administrative process. By fully mobilizing enterprises, social organizations and citizens to participate in crisis response, the government can improve citizens’ satisfaction with public participation, and promote the effective implementation of crisis response policies. By strengthening the publicity of the effectiveness of crisis response policies, the government can enhance the public support for these anti-crisis policy measures and improve the government’s reputation (21, 44).

Moreover, crisis leadership is shaped by government structure and leadership style. First, scholars have shown that governance structures play an important role in crisis response. An effective governance structure can improve leaders’ governance capacity, help them coordinate different organizations and actors to jointly respond to crises (44). Different governance structures (e.g., intergovernmental relations and government organizational structures) lead to different crisis leadership responses (42). For instance, Jae Moon et al. (52) found that the different governance structures of South Korea and Japan drove the two countries to adopt an agile method and a self-restraint-based approach, respectively, in combating COVID-19. A flexible central command and coordination structure is essential to building crisis leadership (53). Crisis leadership also needs to balance centralization and decentralization, clarify the division of responsibilities between departments and facilitate intergovernmental cooperation (16, 36). Thus, a centralized and coordinated system is more conducive to overcoming the bureaucratic fragmentation problem and promoting effective intergovernmental coordination and cooperation to tackle crises.

Second, leadership style shaped by the leader’s personal traits and experiences can affects crisis response by influencing crisis leadership (32). Boin et al. (54) analyzed two elements of the leadership style that includes leaders’ need for control and their sensitivity to context. They found that leaders with strong needs for control adopt a hands-on leadership style to strengthen their control over decision making, while those with weak needs for control adopt a hands-off leadership style to reduce direct intervention in a crisis response. However, effective leadership requires a balance between hands-on and hands-off approaches (55). In terms of leaders’ sensitivity to context, high-complexity leaders (e.g., with a high need for information) are more sensitive and collect information from different sources. Low-complexity leaders are less sensitive and trust members of their inner circles to make decisions (54, 56). Moreover, leaders’ sensitivity to context is affected by their working experience and expertise, with experienced leaders being more likely to gather diverse information and control decision making (57). Therefore, leaders with experience in crisis management are more likely to respond effectively to crises. Political elites’ leadership styles are more conducive to crisis management if they are informed by rich crisis response experience, expertise and high sensitivity to crises.

### Political and social solidarity

Political and social solidarity are shaped by different cultural and social norms. For instance, the political culture of a partisan divide influences public perception of the crisis and can hinder anti-epidemic policy making and implementation (58). In some Asian countries dominated by tight culture, such as China, social cohesion is stronger, and citizens are more likely to obey government policies. However, in some Western countries dominated by loose culture, citizens value more individual freedom (14). Many scholars have shown that trust in the government and health officials is conducive to more effective implementation of public health crisis prevention measures (51). Democracies with higher citizens’ trust in government are more likely to respond effectively to public health crises (19, 59).

This framework examines political and social solidarity from two perspectives, including political polarization and social cooperation with the government. First, political polarization undermines political solidarity and can hamper coordinated action in crisis response (51). Politically polarized democracies are more likely to receive less support for crisis response policies, hindering effective crisis management (60). For instance, political polarization can increase the cost of intergovernmental cooperation and limit the government’s capacity to tackle crises (61). Political polarization also influences the public crisis response. For instance, compared with liberals, conservatives with a lower risk awareness of the epidemic are more suspicious of medical experts and oppose public health measures such as the wearing of masks (62). Additionally, partisans are increasingly distrusting people from opposition parties, and affective polarization is intensifying in the United States (63). People with a high degree of affective polarization are more likely to politicize the issue of scientific epidemic prevention, which can impede crisis response (64). Thus, reducing polarization is conductive to crisis management (65).

Second, social cooperation with the government promotes effective crisis management by enhancing social solidarity. For instance, when citizens abide by anti-epidemic policies, the number of infections and deaths is more likely to be lower (66, 67). Furthermore, the government can use the private sector’s resources and skills to create collaborative crisis management (68). State–society cooperation facilitates policy implementation and co-produces public services during a crisis response. Such social cooperation can be established in different ways. For instance, Moon (69) noted that the government can increase public trust and cooperation by improving information transparency. Tsai et al. (70) found that governments with low social trust can use local intermediaries embedded in communities to strengthen citizens’ cooperation with the government.

Thus, a strong cultural tradition of cooperation between citizens and the government is more conducive to reducing political polarization and social conflicts, thereby enhancing citizens’ trust in the government and improving the government’s capacity to manage crises.

## Methods and data

This article conducts a comparative case study with a most similar system design (71). The US and South Korea have been selected as cases among democratic countries because they are both developed democracies with a very robust healthcare system. However, different contextual factors including individual, institutional and cultural dimensions in the two countries have created different crisis responses by shaping different crisis leadership and political and social solidarity.

First, these two countries are both developed democracies with robust healthcare systems. According to the 2019 Global Health Security Index (72), among 195 countries globally, the US ranked first, and South Korea ranked ninth. This index assesses countries’ capability to maintain health security and prepare for an epidemic. These two countries’ high rankings reflect that they both have a strong capacity to tackle public health crises.

Second, the two countries have responded differently to the current epidemic. Comparing the timelines of crisis responses in the two countries during the early stages of the outbreak can further illustrate the differences in their crisis management. In the United States, on January 2, 2020, the Centers for Disease Control and Prevention (CDC) Director Dr. Robert Redfield and biodefense experts of the National Security Council had raised early warnings of the virus (73, 74). On January 8, President Trump also received an early warning from the intelligence agencies (75). However, President Trump underestimated the severity of the epidemic and did not take timely measures to deal with the crisis. Instead, he tried to downplay the harm of the epidemic (8). For example, on January 22, 2020, President Trump said in an interview with CNBC: “We have it totally under control...It’s going to be just fine” (76, 77). When the World Health Organization (78) indicated that the virus was spreading rapidly among humans on January 22, 2020, the Trump administration also failed to adopt the anti-crisis measures in the pandemic playbook drafted by the Obama administration (79). With the spread of the epidemic, Alex Azar, Secretary of the U.S. Department of Health & Human Services, declared a public health emergency in the United States on January 31, 2020 (80).

In February, 2020, although the CDC had warned that community transmission of the virus had begun (81), the Trump administration still failed to take effective measures in time. It was not until February 26, 2020 that President Trump appointed Vice President Pence to lead the federal government’s response to the crisis (82). President Trump declared a national emergency on March 13, 2020 (83). As cases continued to mount, on March 16, 2020, President Trump finally agreed to take the advice of experts and allow a nationwide 15-day moratorium on nonessential activity to slow the spread of the virus (84). In addition, on March 6, 2020, President Trump promised to make 4 million test kits available by the end of the next week, but only 25,000 tests have been conducted during that time (85). Health and Human Services Secretary Alex Azar has acknowledged that the federal government’s testing system lacks sufficient capacity to deal with the pandemic (86). In the 8 weeks since the first cases were detected in the two countries, the United States has tested only 56,000 people, but South Korea has quickly established a robust testing system and tested 287,000 people (87). Insufficient COVID-19 testing capacity has further exacerbated the spread of the epidemic in the United States.

In South Korea, according to the report *All about Korea’s response to COVID-19* released by the Government of the Republic of Korea, level 1 National Infectious Disease Risk Alert was issued after knowing the cases in China on January 3, 2020. On January 20, 2020, level 2 alert was issued after identifying the first case in South Korea, and the Central Disease Control Headquarters was launched. On January 27, 2020, level 3 alert was issued as cases rise, and the Central Disaster Management Headquarters was launched. On February 23, 2020, level 4 (the highest) alert was issued after Daegu’s massive outbreak, and the Central Disaster and Safety Countermeasure Headquarters was also launched by the Moon administration. Moreover, in early January, 2020, the Moon administration has started promoting the development of COVID-19 testing methods. On February 4, 2020, Emergency Use Authorization was granted for COVID-19 diagnostic reagents. On February 7, 2020, COVID-19 testing was extended to private healthcare facilities across the country. Since March, 2020, the Moon administration has further strengthened border control, the supply of medical supplies such as masks, and the supply of medical services (e.g., the operation of residential treatment centers), so as to effectively treat patients and prevent the spread of the epidemic.

By promptly controlling COVID-19 after the outbreak, South Korea combated the epidemic effectively. In contrast, the Trump administration failed to promptly tackle the crisis, and the number of confirmed cases and deaths soared, making it one of the most severely affected countries globally.

**Figure 2:**
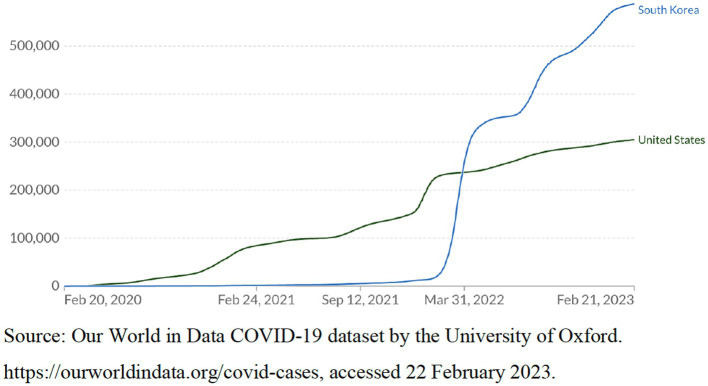
The cumulative confirmed COVID-19 cases per million people, as of February 21, 2023.

**Figure 3:**
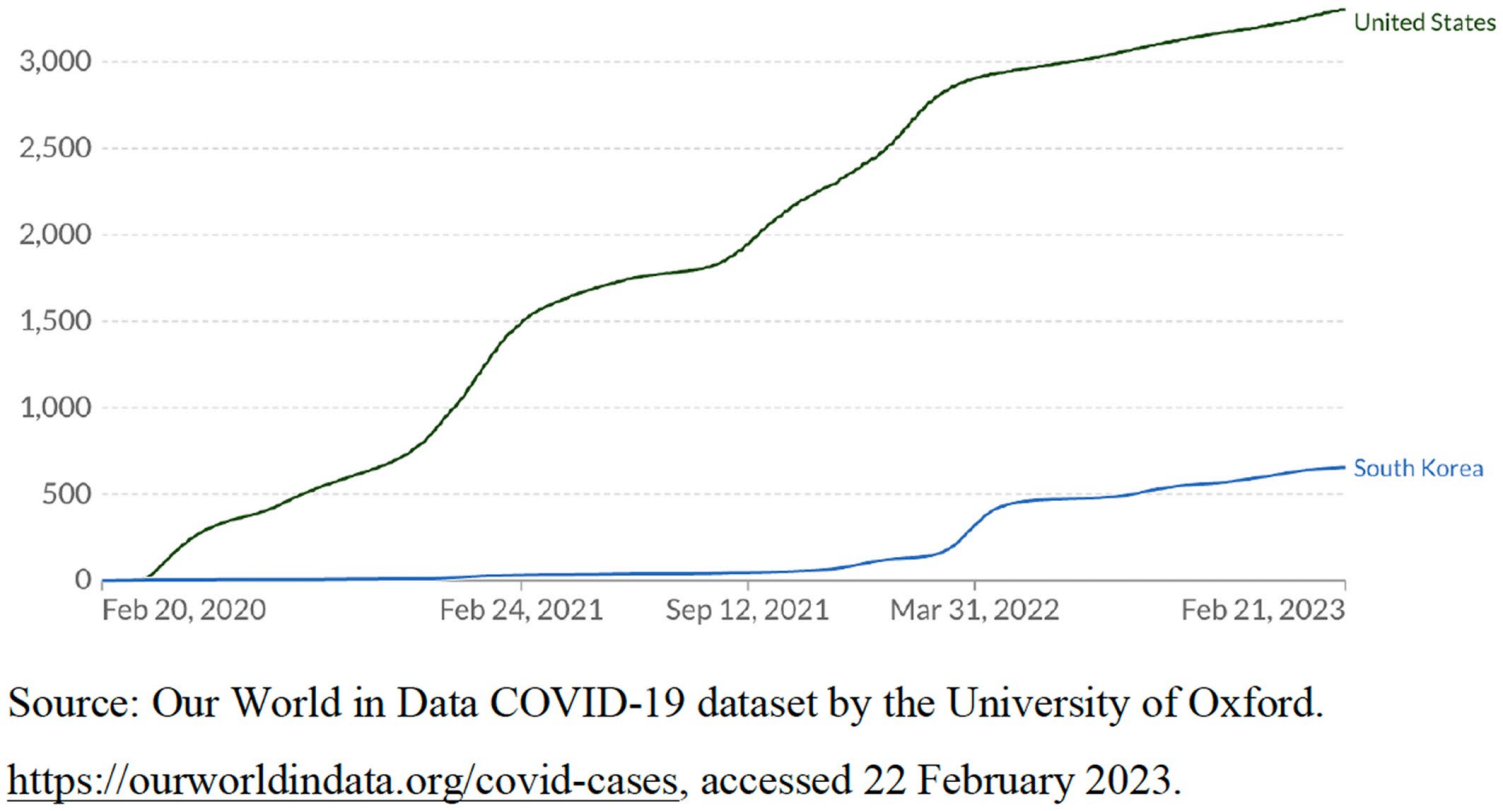
The cumulative confirmed COVID-19 death per million people, as of February 21, 2023.

**Figure 4:**
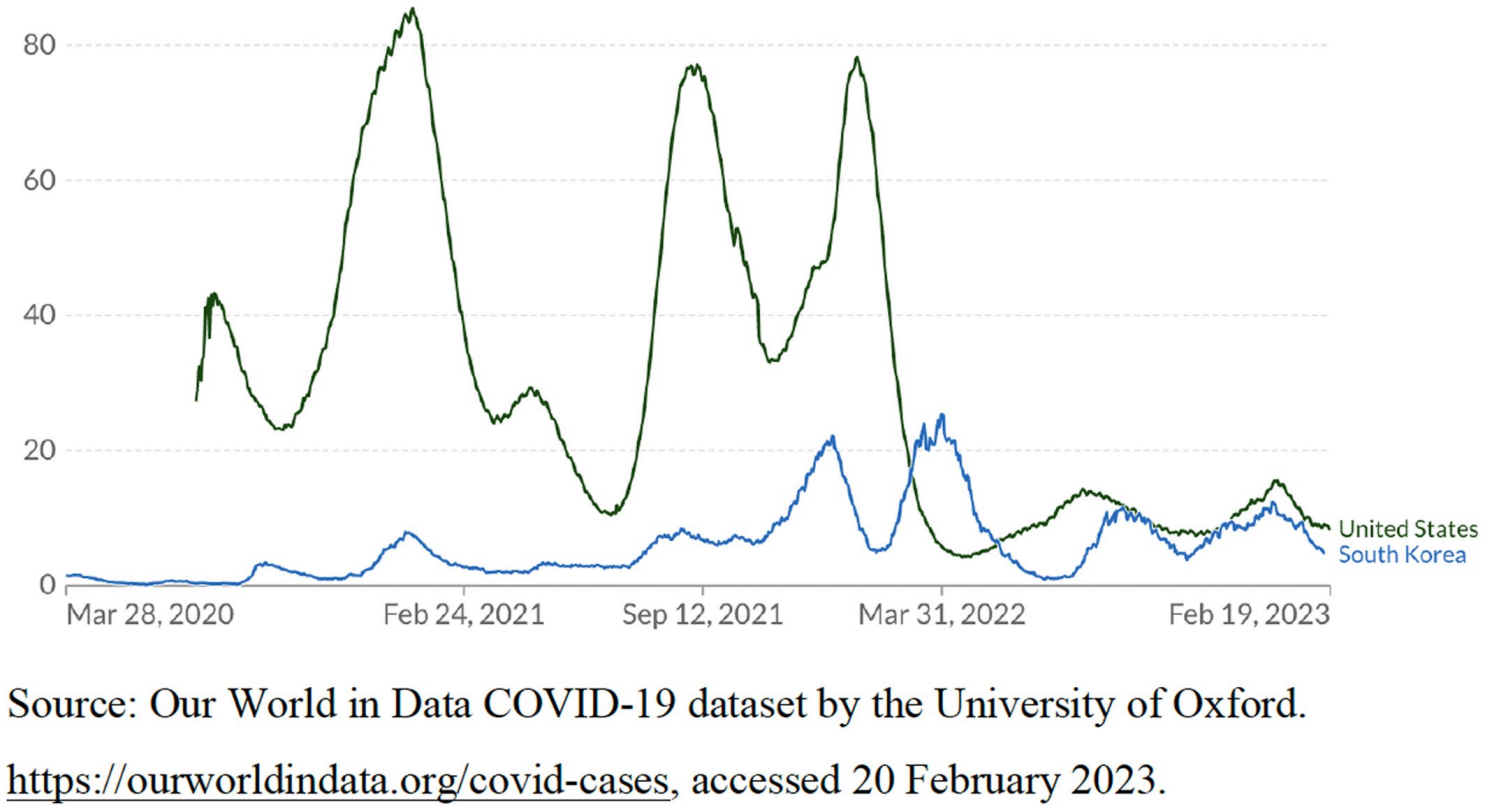
The number of COVID-19 patients in intensive care (ICU) per million people, as of February 19, 2023.

According to the World Bank database (88), as of 2022, the population of the United States is about 333 million, with a *per capita* GDP of $76,398.6. The population of South Korea is about 51.6 million, with a *per capita* GDP of $32,254.6. According to government statistics from both countries (89, 90), in 2022, the population of the United States and South Korea aged 65 and above was about 57.8 million and 9 million, respectively, accounting for approximately 17% of the total population of both countries. The population of the United States is much larger than that of South Korea. Considering the different populations of the two countries, we select different indicators to compare their effectiveness in responding to the crisis. These indicators include these two countries’ statistics on the cumulative confirmed COVID-19 cases per million people, the cumulative confirmed COVID-19 deaths per million people, and the number of COVID-19 patients in intensive care per million people from 2020 to 2023. In 2020 and 2021, these statistics of the United States were far higher than those of South Korea. Figures 2–4 show these indicators.

Since 2022, considering that the death rate of the virus has dropped significantly and the vaccine coverage in the population has become high, in order to promote economic recovery and social development, South Korea has gradually relaxed strict anti-crisis measures, which led to a sharp increase in the number of confirmed cases (see Figure 2) (91). However, Figure 3 demonstrates that the number of confirmed COVID-19 deaths per million people in South Korea is still far lower than that in the United States. According to the WHO COVID-19 database (92), as of February 21, 2023, the cumulative confirmed COVID-19 deaths per million people in South Korea was about 66, far lower than that of most democratic countries. On the contrary, the cumulative confirmed COVID-19 deaths per million people in the United States was about 333.5, far higher than that in most other democratic countries.

Furthermore, according to Bloomberg’s Covid Resilience Ranking updated on June 29, 2022, among 53 economies with more than $200 billion in the world, South Korea’s resilience score ranked first, and the United States ranked 36 (93). This demonstrates South Korea’s effective and resilient crisis response, which is conducive to reopening borders and restoring economic growth with a low death toll, thereby reducing the damage to business and social development caused by the epidemic response.

In addition, studies have regarded the United States and South Korea as typical cases of poor and effective response to the epidemic in democratic countries (94). Scholars have also conducted comparative case studies on the different responses of the United States and South Korea to the epidemic (20, 21, 95–98). Therefore, a comparison of these two cases allows us to explain different crisis responses in democracies with similar healthcare capacities.

Third, the two countries have different contextual factors including the leadership style, governance structure and cultural and social norms based on individual, institutional and cultural dimensions, respectively. These factors influence different crisis responses by shaping different crisis leadership and political and social solidity in the two countries. For example, in the United States, President Trump was not sensitive to crisis information and lacked experience in crisis response. He underestimated the severity of the epidemic. Meanwhile, the Trump administration damaged the crisis management frameworks and ignored the scientific opinions and early warning of experts (99). Under the federalist system, poor coordination between the federal government and state governments also weakened the crisis leadership. The serious political polarization and the weak social norm of cooperating with the government in the United States have further weakened the political and social solidarity. These factors together hinder effective crisis response.

In South Korea, President Moon has experience in crisis response, strong sensitivity to crisis, and attaches great importance to expert opinions and crisis response. Moreover, South Korea has established an effective information collection and crisis early warning system, as well as a centralized public health management system and intergovernmental communication and coordination mechanism to strengthen crisis leadership. In addition, the political polarization of South Korea is weaker than that of the United States, and the strong norm of social obedience to the government has enhanced political and social solidarity. These factors jointly promote effective crisis response. Therefore, the comparative case study of the United States and South Korea can explain how different contextual factors affect the crisis response of democratic countries.

This study collected data from different sources, including government documents, official websites, leaders’ speeches, research reports, academic articles and news media. First, we collected the two countries’ government policy documents and leaders’ speeches on epidemic response, and some authoritative documents from CDC and other professional institutions for public health crisis response. For example, these documents include remarks by South Korean President Moon Jae-in, Prime Minister Chung Sye-kyun, *All about Korea’s response to COVID-19* released by the Government of the Republic of Korea, *Korea’s evolving response to COVID-19* (5th ed.) released by the Ministry of Foreign Affairs, Republic of Korea, remarks by U.S. President Donald Trump and Vice President Mike Pence from the White House, *“It was Compromised”*: *The Trump Administration’s Unprecedented Campaign to Control CDC and Politicize Public Health During the Coronavirus Crisis* released by the Select Subcommittee on the Coronavirus Crisis established by the House of Representatives, *Characteristics of health care personnel with COVID-19 - United States* released by the CDC COVID-19 Response Team. We also checked the governments’ official websites of the two countries, these authoritative documents truly record how the two countries responded to the crisis.

Second, we collected data from the World Health Organization and other authoritative research institutions, such as WHO COVID-19 database, Our World in Data COVID-19 dataset released by the University of Oxford, Bloomberg’s Covid Resilience Ranking, 2019 *Global Health Security Index Report* released by Johns Hopkins Center for Health Security, Nuclear Threat Initiative and Economist Intelligence Unit. These data can help us further understand the public health crisis management capabilities of the United States and South Korea and their different performances in crisis response, so as to answer our research questions more effectively.

Third, we also collected data on how the two countries responded to the crisis from authoritative media with credibility, such as The Washington Post, The New York Times, The Wall Street Journal, BBC News, Reuters, and CNBC (Consumer News and Business Channel). The data reliability of these well-known media is relatively high, which can further support our case analysis. We also watched some TV programs (e.g., Reuters, CNN, CBS News, Bloomberg News, Arirang News and KTV National Broadcasting System from South Korea) on the response to the epidemic in the two countries, including dialogues and interviews with some government leaders and public health experts, and their speeches. Government leaders include U.S. President Trump, Vice-President Pence, Health and Human Services Secretary Alex Azar, other Trump Administration officials, South Korean President Moon Jae-in, Prime Minister Chung Sye-kyun, and Foreign Minister Kang Kyung-wha etc.

From these media reports, we also collected interviews and opinions from infectious disease and public health experts, such as Dr. Anthony Fauci, the Director of the National Institute of Allergy and Infectious Diseases, Dr. Robert R. Redfield, the CDC director, Dr. Nancy Messonnier, director of the CDC’s National Center for Immunization and Respiratory Diseases, Dr. Eun-Kyeong Jung, the director of the Korean Centers for Disease Control and Prevention (KCDC), and Yong-Kyun Kim, the director-general of South Korea’s National Disaster and Safety Control Center. These experts assisted the government in fighting the epidemic by providing expertise and scientific advice. They have a good understanding of how the two countries responded to the crisis, which can provide valuable evidence for our research. By using diversified data sources, we were able to enhance the reliability of evidence and show more details about how the two countries fought COVID-19.

Furthermore, we attended some webinars on the two countries’ responses to COVID-19 such as “Managing ongoing surges—lessons from the front lines” organized by the National Academy of Medicine and American Public Health Association on 29 July 2020, “The pandemic puzzle: lessons from the U.S. response to COVID-19″ organized by Stanford University School of Medicine and Stanford Graduate School of Business on 17 September 2021, Korea Policy Forum Webinar “South Korea’s Response to the Coronavirus” organized by the George Washington University Institute for Korean Studies on April 23, 2020, “COVID-19 webinar: what can we learn from South Korea’s response?” organized by Korean Connection on 24 November 2020, “What made South Korea’s COVID-19 response so successful?” organized by Harvard Ash Center on December 3, 2020, and “COVID-19 in Public Management and Policy Research: Challenges and Prospects” organized by the Department of Public Policy, City University of Hong Kong on 17 September 2021.

From these webinars, many of the two countries’ experts and officials involved in the response to the crisis provided a lot of valuable information. They include Rochelle Walensky, the chief of the Infectious Diseases Division at Massachusetts General Hospital and a professor from Harvard Medical School, Milana Boukhman, Clinical Professor of Emergency Medicine, Stanford Medicine, Howard Zucker, New York State Health Commissioner, Mandy Cohen, Secretary of North Carolina Department of Health and Human Services, Yong-Kyun Kim, the director-general of South Korea’s National Disaster and Safety Control Center, Chang Huh from the South Korean Ministry of Economy and Finance, Hee-Kwon Jung from the South Korean Ministry of Science and ICT, Moran Ki from the South Korean National Cancer Center Graduate School of Cancer Science and Policy, and Professor M. Jae Moon from Yonsei University, South Korea etc.

We can collect data from the sharing of the two countries’ government officials, scholars and medical experts on crisis response. Due to the epidemic and travel restrictions, we did not conduct extensive field research in the two countries. However, these data can strengthen the research’s credibility, reflect the true situation of the two countries’ crisis responses, and further examine our analytical framework.

In addition, we also collected and reviewed many articles on the COVID-19 responses in the United States and South Korea published in well-known academic journals across disciplines such as public health, public administration and political science. These data can also provide reliable data for us to answer our research questions.

Although we tried to enhance the reliability of the data by comparing different data sources to analyze the epidemic response of the two countries, we acknowledge that our data may have weaknesses, such as possible bias in secondary sources.

## Results

### How crisis leadership shaped by leadership style and governance structure affects crisis response in the US and South Korea

This section demonstrates how different leadership styles and governance structures affect crisis response in the two countries by shaping different crisis leadership. We compare different crisis leadership in the US and South Korea from three capacities: sense making, decision making and coordinating, and meaning making (see Table 1).

**Table 1:** A comparison of crisis leadership in containing COVID-19 in the US and South Korea.

	United States	South Korea
Sense making capacity	Weak	Strong
Decision making and coordinating capacity	Weak	Strong
Meaning making capacity	Weak	Strong

#### Sense making

The Trump administration demonstrated a weak sense making capacity and failed to timely detect the crisis by collecting and analyzing information. In terms of leadership styles, without any experience in tackling public health crises, President Trump was insensitive to coronavirus warnings and overconfident in dealing with the crisis (8). He underestimated the severity of the crisis and did not deal promptly with epidemic information. The CDC had detected the virus as early as January, and established an agencywide response to the crisis on January 21, 2020 (100). Some public health experts in the US have also issued warnings of the crisis in the early stage of the outbreak (101, 102).

However, there is a serious cognitive divide between experts and political leaders especially President Trump (21). The Trump administration did not adopt these expert opinions, seriously underestimated the risk of the epidemic, and failed to timely take effective measures to deal with the crisis (e.g., giving early warning to the public) after the outbreak (99). For example, after CDC experts discovered that the epidemic was spreading on a large scale in Europe and issued a warning, the Trump administration did not quickly take travel restrictions on travelers from Europe, resulting in a large number of European travelers entering the US, which exacerbated the risk of the epidemic in the US (103). On February 26, 2020, Dr. Nancy Messonnier, director of the CDC’s National Center for Immunization and Respiratory Diseases warned that the virus would spread in American communities. But President Trump still described the virus as “a problem that’s going to go away” (104). President Trump even threatened to remove Dr. Nancy Messonnier over her warnings about the risks of the pandemic (86).

In terms of governance structure, the Trump administration damaged the crisis management frameworks. Namely, in 2018, the Trump administration abolished the National Security Council Directorate for Global Health Security and Biodefense established by the Obama administration, so the US public were not alerted in time about the COVID-19 outbreak (105). After the National Security Council Directorate for Global Health Security and Biodefense was abolished by the Trump administration, there is no other professional organization to assume this responsibility, and the National Security Council has not effectively played the role of crisis warning (99). Dr. Anthony Fauci, a well-known infectious disease expert, stated that “It would be nice if the office was still there” (106). Similarly, Beth Cameron, the first director of the agency, said in relation to this:

I was mystified when the White House dissolved the office, leaving the country less prepared for pandemics like COVID-19. The U.S. government’s slow and inadequate response to the new coronavirus underscores the need for organized, accountable leadership to prepare for and respond to pandemic threats … When this new coronavirus emerged, there was no clear White House-led structure to oversee our response, and we lost valuable time (107).

President Trump also defunded the Pandemics and Emerging Threats Office established by the Obama administration, thereby weakening the federal government’s preparedness and response to the outbreak (108).

In contrast, the South Korean leader’s experience in tackling public health crises, along with a robust professional governance structure, enabled the Korean government to have a strong sense making capacity. In terms of leadership styles, President Moon has rich experience in crisis response and remains very alert to crises. For instance, when MERS broke out in 2015, Moon Jae-in, as the then leader of the opposition party, strongly criticized the central government for failing to quickly disclose epidemic information and respond to the epidemic, and he also proposed policy recommendations for crisis management (109).

Based on this experience, in terms of governance structure, President Moon emphasized information collection and crisis warning in the early stage of the epidemic. For example, after the MERS crisis, the public health crisis response system was reconstructed. The role of the KCDC in crisis management was enhanced, which enabled it to collect and disclose timely information in January 2020 during the early stage of the epidemic (69). For instance, the government issued the first-level National Infectious Disease Risk Alert after knowing the cases in China on January 3, 2020. On January 20, 2020, the government issued the second-level alert after identifying the first case in South Korea, and launched the Central Disease Control Headquarters. The Moon administration further raised the alert to the fourth-level (the highest level) after Daegu’s massive outbreak on February 23, 2020 (110).

In September 2020, President Moon promoted the KCDC to the Korea Disease Control and Prevention Agency (KDCA), giving it a higher status with more autonomy and resources (111). The government also established a national information management system that facilitates information collection and sharing to detect crises (110). In addition, South Korea has also established the National Committee for Clinical Management of Emerging Infectious Diseases to promote information sharing among medical staff and use their expertise to strengthen patient care (110).

#### Decision making and coordinating

The Trump administration’s inadequate decision making and coordinating capacity weakened its crisis leadership. Before President Trump took office, he was a businessman and lacked experience and capacity in government decision making and coordination. He did not adopt expert advice to make scientific decisions to contain COVID-19. For example, President Trump appointed people who lacked professional capabilities to manage crisis response agencies, which undermined the federal government’s crisis response system and the credibility of emergency management agencies (e.g., the Federal Emergency Management Agency and the CDC) (99). Public health experts such as Dr. Anthony Fauci were marginalized. Their suggestions, such as encouraging citizens to wear masks (a non-pharmacological intervention), were not adopted (112).

In terms of governance structure, a lack of coordination between federal and state governments can hinder crisis management. The lack of national crisis leadership prevents the Trump administration from effectively coordinating the states’ crisis responses (e.g., production, sales, and distribution of medical supplies) (27). The Trump administration did not coordinate and cooperate with state governments. For example, instead of effectively coordinating the procurement and distribution of medical equipment among the states, the Trump administration competed with states to obtain these equipment, but ultimately failed to effectively distribute these equipment to where it is most needed (28). Furthermore, the Trump administration shirked the responsibility of epidemic control to state governments and did not provide adequate assistance to them, which made it more difficult to contain COVID-19. For instance, due to a lack of the Trump administration’s support, California’s response to the epidemic was weakened (108). On March 19, 2020, President Trump said that “The federal government is not supposed to be out there buying vast amounts of items and then shipping. You know, we are not a shipping clerk” (113). President Trump also told Vice President Pence not to call governors who do not ‘appreciate’ White House crisis response in March 2020 (114).

Moreover, the White House COVID-19 task force only focused on how to respond to outbreaks and lacked a systematic long-term plan for epidemic testing, tracking and emergency medical supplies (115). A serious shortage of medical supplies such as face masks and test kits had caused a large number of medical staff to be infected in the early stage of the crisis. According to the CDC report, as of April 9, 2020, more than 9,000 medical staff had been infected (116).

In addition, the Trump administration has also intervened in the operation of the CDC, weakening the CDC’s role in decision making. For example, Deborah Birx, coordinator of the White House Coronavirus Task Force, opposed the CDC’s guidelines and did not respect the CDC. In a task force meeting in May 2020, she once said, “There is nothing from the CDC that I can trust” (117). The Trump administration also undermined the role of the CDC in crisis response by putting pressure on CDC experts who told the truth and manipulating CDC publications. For example, CDC Director Dr. Robert R. Redfield admitted that Trump administration officials have interfered with CDC’s guidance documents (118). On July 23, 2020, the CDC relaxed its guidelines on school reopening after President Trump criticized its previous guidance (119).

In contrast, President Moon once served as President Roh Moo-hyun’s chief of staff, which helped him accumulate rich experience in government decision making, coordination and social communication (120). Under President Moon’s leadership, the central government has learnt the MERS lessons, adopted the public health experts’ suggestions and made timely and scientific decisions to control the epidemic. For example, after the outbreak of the epidemic, the Moon administration fully respected the opinions of experts, and flexibly adjusted different levels of social distancing policies based on the scientific analysis of the severity of the epidemic by epidemiologists and medical experts (110). Meanwhile, following the recommendations of the medical community, the Moon administration established the drive-through and walk-through screening stations to improve testing efficiency and reduce the risk of infection among medical staff (121). Scientific decision-making based on expertise has effectively prevented the spread of the virus and minimized the negative impact of epidemic prevention policies on economic and social development. Public health experts such as Dr. Jung Eun-kyeong (the KCDC director) have made significant contributions to the government’s decision making. Dr. Jung Eun-kyeong was praised by President Moon as an anti-epidemic hero and was appointed as the first KDCA Commissioner in September 2020 (111).

In terms of governance structure, South Korea’s centralized public health management system has played a pivotal role in containing COVID-19. The central health department and KCDC can guide local crisis management effectively, promote the establishment of a unified anti-pandemic system in various regions and avoid loopholes in epidemic prevention (122). In this process, a flexible and effective intergovernmental coordination mechanism has been established by strengthening intergovernmental communication and by involving local leaders in the central epidemic prevention meeting (110). For instance, after the COVID-19 outbreak in Daegu, the national alert was raised to the highest level starting from February 23, 2020. South Korean Prime Minister Chung Sye-kyun hosted the Central Disaster and Safety Countermeasure Headquarters Meeting. The participants of this meeting mainly included those from the central government epidemic prevention related departments, as well as officials from 17 provinces and major cities. The Prime Minister chaired the meeting almost every day from February 23, 2020 until late April, 2020 (110).

This intergovernmental coordination mechanism enables the central government to coordinate different local governments, integrate and allocate resources and promote intergovernmental cooperation in crisis management. For example, according to the report *All about Korea’s response to COVID-19* released by the Government of the Republic of Korea (110), after a large-scale outbreak in Daegu in February 2020, the number of cases surged, which put a lot of pressure on local medical institutions. The central government coordinated with local governments and medical institutions in other surrounding provinces or cities in a timely manner, so that patients could be transferred to these places in time for treatment. In June 2020, the Central Disaster and Safety Countermeasure Headquarters also integrated the medical resources from 17 provinces and cities into 6 regional clusters, which can help eliminate bureaucratic barriers when transferring patients across regions, so as to provide better service for patients. In addition, according to the sharing from Yong-Kyun Kim (the director-general of South Korea’s National Disaster and Safety Control Center) in the webinar “What made South Korea’s COVID-19 response so successful?” organized by Harvard Ash Center on December 3, 2020, President Moon fully empowered Dr. Jung Eun-kyeong (the KCDC director) to lead other health experts to participate in epidemic prevention, so that the KCDC can play a greater role in the scientific decision-making of crisis response.

#### Meaning making

The Trump administration’s weak meaning making capacity impeded crisis communication and management. The government did not provide accurate epidemic information or communicate effectively with its citizens, which misled the citizens’ perception of COVID-19. For example, in the early stage of the crisis, the Trump administration declared that the epidemic was under control and that the virus was far away from the US (112). Although President Trump privately admitted that he knew that the virus had spread in the community, he claimed in February 2020 that the epidemic was under control, misleading the government and citizens about the crisis (20). The political motivation to maintain economic growth and win the re-election has resulted in politics and cognitive biases overriding scientific decision-making in the federal government and crisis communication with citizens, seriously affecting the crisis response in the US (74, 123). Due to this misjudgment of the crisis, citizens relaxed their vigilance regarding the crisis, thereby intensifying the spread of the epidemic.

President Trump also misled citizens by not adopting appropriate public health measures. For example, he participated in large-scale gatherings without wearing face masks, which further misled the public, hindered effective anti-crisis policy implementation (112). Moreover, President Trump’s crisis communication had anti-government characteristics. He doubted the bureaucracy’s role in crisis response and criticized Democratic governors who adopted epidemic control measures such as shutdowns (99). Such pronouncements weakened the government’s authority, crisis leadership and anti-epidemic policy implementation.

In addition, in terms of governance structure, there are fragmentation problems arising from anti-epidemic policies between the federal and state governments and between different state governments, resulting in inconsistent epidemic prevention policies in various regions (124). Such inconsistent epidemic prevention policies aggravate social conflicts and distrust of the government, thereby exacerbating the crisis.

In contrast, in terms of leadership style, South Korea’s President Moon learned from the failure of the MERS response in 2015 by emphasizing transparency, civic participation, and crisis communication with the South Korean citizens. For example, after cases were detected in January 2020, to make citizens vigilant and enhance their awareness of self-protection, the government quickly issued a warning and raised the national alert level to the highest in February 2020 (125). As President Moon said at the G20 2020 Extraordinary Virtual Leaders’ Summit on March 26, 2020:

The time is never right for complacency, yet preemptive and transparent infectious disease prevention and control measures, combined with the public’s voluntary and democratic participation in such efforts, are bringing gradual stability (126).

In terms of governance structure, the Moon administration has established an effective crisis communication mechanism, so that citizens can obtain timely epidemic information and meet their needs for medical supplies. For instance, the South Korean government held press briefings twice a day (110). With the strong support of President Moon, Dr. Jung Eun-kyeong (the KCDC director) led other health experts to contain the COVID-19 pandemic and hold daily press conferences in which they delivered transparent epidemic information to citizens, and issued warnings on the risks of the epidemic to remind the public to be well prepared for the crisis (127). By cooperating with enterprises and citizens, the government used information technology to develop mobile apps to disclose information and strengthen self-health checks (121). Once a confirmed person is found, the local government can immediately send information to this patient and send the risk-warning message about the places that the patient visited to other citizens’ mobile phones.

In addition, through cooperation with agencies such as the Korea Communications Commission, the Cyber Bureau of the Korean National Police Agency requests police investigators to track down false information on the Internet and social media that hinders epidemic prevention and to remove false news that misleads citizens’ perceptions and causes social unrest (110).

### How political and social solidarity shaped by cultural and social norms affects crisis response in the US and South Korea

This section shows how different cultural and social norms affect crisis management in the US and South Korea by shaping political and social solidarity. Political and social solidarity are measured by political polarization and social cooperation, respectively (see Table 2).

**Table 2:** A comparison of political polarization and social cooperation in the US and South Korea.

	United States	South Korea
Political polarization	High	Medium
Social cooperation	Weak	Strong

#### Political polarization

A high degree of political polarization rooted in American political culture exacerbates the division of people with different ideologies (e.g., Democrats and Republicans), making cooperation between parties difficult in crisis management (99). In recent years, Americans have increasingly distrusted people from other parties, and hostility between different parties has deepened (63). Citizens with different political stances demonstrate different perceptions of COVID-19, and it is difficult to reach a consensus. States led by governors from different parties also take different anti-epidemic measures. For example, some Democrat-led states such as California issued stay-at-home orders, but eight states where Republicans are governors had not conducted this policy as of April 22, 2020 (124). Consequently, the inconsistency and fragmentation of anti-epidemic policies between different regions has hindered the crisis response (21).

Moreover, political polarization politicizes scientific public health measures, which can hamper crisis management. For example, the CDC’s proposal for citizens to wear face masks and maintain social distancing was opposed by President Trump and some right-wing Republicans. President Trump has also politicized and criticized effective public health interventions such as the policy of wearing face masks conducted by some states, which not only weakened the federal government’s effective response to the crisis, but also exacerbated the spread of the virus (28). The “Fire Fauci” campaign against Dr. Fauci, who advocated anti-epidemic measures, appeared on Twitter in April 2020 and was reposted by President Trump (128). Moreover, research has found that regions that voted for President Trump have lower compliance with mobility restrictions (129).

South Korea also faces political polarization (130). The ruling party’s anti-epidemic policy is criticized by opposition parties and religious groups. For example, some conservative religious groups and Christians were unwilling to maintain social distancing. These groups still organized large-scale gatherings and criticized the anti-epidemic measure to ban such gatherings as a means by which President Moon could reduce people’s protests (131). Many people wanted to impeach President Moon through online petitions, but many people also petitioned to support President Moon, which shows the division in society (132).

However, South Korea’s political polarization is not as serious as that in the US. The main reason for this is that after South Korea’s transition from authoritarianism to democracy, the government still has a strong crisis management capacity (133). Despite criticisms, the Moon administration employs a strong state capacity to take measures to control COVID-19 and does not allow the epidemic prevention work to stall because of different partisan debates. The government’s effective crisis responses have gained public support, which helped enable the ruling party to win the parliamentary elections in April 2020 (134). Thus, strong government crisis leadership can overcome political polarization and enhance social cohesion in a crisis response. In contrast, the US political cultural tradition is characterized by intense partisan divergence, and the federal government’s ability to control political polarization is therefore weaker.

#### Social cooperation

Drawing on the theory of cultural tightness–looseness (the strength of social norms and the degree of sanctioning within societies) from Gelfand et al. (135), we analyze how the different cultures of the US and South Korea shape different social cooperation, which in turn affects crisis management in the two countries. According to the definition of Gelfand et al., loose culture refers to “culture that have weak norms and a high tolerance for deviant behavior,” and tight culture refers to “culture that have strong social norms and a low tolerance of deviant behavior” (136, 137). Michele J. Gelfand et al. used this theory to divide the different cultures of different countries, and found that the US has individualistic and loose cultural characteristics, including a weaker social norm and more tolerance for deviant behavior. South Korea has collectivistic and tight cultural characteristics, including a strong social norm and less tolerance for deviant behavior (138, 139).

Based on these existing studies, we use loose culture and tight culture to describe the cultural characteristics of the US and Korea, respectively. We compare the overall cultural differences between the two countries at the national level, and many studies have supported this comparison (51, 137, 139). The loose and tight cultures of different countries have also had an important impact on social cooperation in crisis management.

Specifically, the US is dominated by individualism. Indeed, the US political system’s core value is to protect individual freedom and restrict government intervention (140). Due to its loose culture, however, the US has a weak social norm of citizen obedience to the government (141). The protection of personal freedom and private information limits the US government’s ability to control its society by enforcing compulsory anti-epidemic measures. Additionally, the US risks a decline in social capital (142), which could exacerbate social non-compliance with anti-epidemic policies.

Some US states adopted anti-epidemic measures such as stay-at-home orders, which affect citizens’ daily lives and cause public dissatisfaction. A large number of anti-lockdown protesters appeared in some states [BBC (143)]. Many residents argued that these anti-epidemic measures are overly severe, threatening their personal freedom, and seriously affecting their lives and work. The government cannot force them to implement these measures.

However, citizen protests against the epidemic prevention measures have increased the risk of the epidemic spreading. Moreover, partisan prejudice, suspicion of science and a lack of civic obligations—embedded in the American political culture and social norms—further hinder government–society cooperation in combating COVID-19 (140).

In addition, we acknowledge that due to its large size, larger population, and heterogeneity, the US population does not always share the same beliefs and behaviors, particularly in comparison to more homogenous societies like South Korea. For instance, cultural tightness–looseness also varies at the state level in the US (139, 144). Existing studies have shown that regional crisis responses are heterogeneous (43, 145). Considering the complexity of the internal governance system of democratic countries and the cultural differences in different regions, future research can further analyze why different regions within a democratic country such as the US respond differently to crises.

In contrast, South Korea is characterized by tight culture, with a social norm of state–society cooperation, which facilitates effective crisis response (141). President Moon said in relation to this at the 73rd World Health Assembly in May 2020:

The Korean people displayed the highest form of civic virtues to practice the spirit of ‘freedom for all’ and voluntarily participated in quarantine efforts. This was what really enabled the three main principles of openness, transparency, and democracy to flourish (146).

Most South Korean citizens support the government’s anti-epidemic policies. Citizens’ voluntary obedience promotes effective crisis management (69). A large number of volunteers even participated in epidemic prevention. For example, from January 20 to March 17, 2020, the number of volunteers involved in caring for patients and serving the community exceeded 180,000 (21). Civic organizations also cooperate with the government and medical institutions to provide assistance such as daily necessities for vulnerable groups and quarantined people (147).

Additionally, public–private partnerships contribute to crisis response. For example, with the aid of government mobilization, enterprises have cooperated with the government to accelerate the production of medical supplies such as test kits. In turn, the government has been quick to grant enterprises emergency-use authorizations for production. The government–enterprise cooperation has greatly improved the ability to detect viruses. Enterprises have also used advanced technology to help the government develop mobile apps to control the epidemic, such as an app for tracking the trajectory of confirmed cases (110).

## Discussion

The COVID-19 pandemic has posed a serious threat to the governance of democracies (3). Different democracies have responded to the pandemic in different ways and have showed different crisis management performance (6, 13). Some democratic countries can quickly collect information, promptly monitor public health crises, and take measures to manage the crisis (2). However, some democracies have failed to deal with the crisis in a timely manner (99). This article focuses on why democracies respond to crises differently and how different contextual factors affect crisis responses in democracies.

Studies have found that contextual factors play an important role in crisis response in democratic countries. For example, scholars have discussed that the degree of democracy (18), protection of basic democratic principles (5), different welfare systems (24), and citizens’ trust in government (19, 59) have important effects on crisis response in democracies. The relationship between the administrative department and the legislature (23), and intergovernmental relations such as different federal systems also shape crisis management in democracies (7, 16, 17).

However, the existing research lacks a systematic analytical framework to examine the specific mechanism of different contextual factors affecting crisis management in democratic countries. How different contextual factors affect crisis response in democracies is underexplored. The purpose of this study is to fill this gap and contribute to research on public health crisis management in democracies through a comparative case study of COVID-19 responses in the US and South Korea.

First, this article proposes a context-contingent integrative framework to analyze the specific mechanism of contextual factors influencing public health crisis management. We find that contextual factors including individual, institutional and cultural dimensions can influence crisis management in democratic countries by shaping crisis leadership and political and social solidarity. Specifically, on the institutional and individual dimensions, the governance structure and politicians’ leadership styles jointly shape crisis leadership, which in turn affects crisis response. On the cultural dimension, cultural and social norms can influence crisis response by shaping political and social solidarity.

Crisis leadership and political and social solidarity are two key factors that affect crisis response. This framework focuses on sense making, decision making and coordinating, and meaning making in crisis leadership, and shows how these capabilities can contribute to public health crisis management. For example, the sense making capacity helps the government collect and analyze information to detect crises in a timely manner and issue early warnings. The decision making and coordinating capacity enables the government to quickly formulate anti-crisis measures after a crisis occurs, and coordinate different organizations to jointly respond to the crisis. The meaning making capacity can help the government strengthen crisis communication with citizens, enhance social trust and support for the government, and enhance the legitimacy of crisis governance. Political and social solidarity are measured by political polarization and social cooperation, respectively. Strong social cooperation and a low level of political polarization can promote cross-organizational cooperation and reduce the cost of policy implementation, thereby enabling effective public health crisis management.

Furthermore, these findings also enhance our understanding of crisis leadership and political and social solidarity in crisis response in democracies. Existing research mainly emphasizes the significance of crisis leadership and political and social solidarity to crisis management (2, 31, 36). This article further develops the theories of crisis leadership and political and social solidarity by revealing how contextual factors based on the three dimensions affect crisis leadership and political and social solidarity in public health crisis management.

On the individual dimension, rich crisis management experience, expertise, and greater crisis sensitivity can shape the leadership style of political actors, making them more likely to have stronger and more resilient crisis leadership to respond effectively to crises. On the institutional dimension, a governance structure characterized by an effective centralized and coordination system can promote cross-organizational collaboration (e.g., intergovernmental and interdepartmental collaboration, and public-private partnerships), thereby enhancing government crisis leadership to effectively manage crises. On the cultural dimension, tight culture and a tradition of strong social cooperation with the government are conducive to strengthening political and social solidarity by reducing political polarization and enhancing social cohesion and public support in anti-crisis policies, so that the government and society can work together to manage public health crises.

## Conclusion

By comparing different COVID-19 responses in the US and South Korea, this research has explored why democracies respond differently in crisis management. By proposing a context-contingent integrative framework for public health crisis management, we find that different contextual factors including individual, institutional and cultural dimensions can influence different public health crisis responses in democracies by shaping crisis leadership and political and social solidarity. We seek to achieve analytic generalization rather than statistical generalization to generalize the findings from our study (148). In addition to the US and South Korea, our analytical framework can also be used to analyze how other democracies with different contextual factors respond to crises.

This research provides valuable insights for enhancing democratic resilience and achieving effective public health crisis management in democracies. First, establishing a robust healthcare system is generally considered to be the key to tackling the public health crisis. However, this advantage is likely to make the government overconfident in its crisis management capacity, underestimate the risk of an epidemic and fail to take timely measures when crises occur (13). This article shows that it is not enough to rely solely on the healthcare system. The crisis leadership of the government, as well as political and social solidarity, plays a key role in crisis response.

For example, although the US has the world’s top health security system, the Trump administration’s crisis leadership in the early stage of the outbreak was highly ineffective and even counter-productive. President Trump politicized the epidemic and failed to take timely scientific anti-epidemic measures, which led to the spread of the virus. Moreover, the high degree of political polarization (e.g., partisan conflicts) in the US and the weak social norm of social cooperation with the government further hindered epidemic control. Additionally, from the Trump administration’s early missteps (e.g., underestimating the crisis and spreading the misperception that COVID-19 is a “mild flu”) in its response to the crisis, we can also find that populism, fake news and demagogic policies may compromise not only the response to the epidemic but the pillars of democracy itself.

Second, countries’ different epidemic responses have ignited fierce debates about whether democracy or authoritarianism is more effective in managing crises (2). Democracies have been facing challenges such as democratic backsliding in the past few years (149). The mistakes made by some democracies in tackling COVID-19 have further impacted on people’s confidence in democratic governance. This research shows that democratic countries need certain conditions to manage crises and reinvigorate democracy. Democracies with a strong crisis leadership and a high degree of political and social solidarity are more likely to tackle public health crises effectively. Crisis leadership and political and social solidarity are shaped by different contextual factors including individual, institutional and cultural dimensions (e.g., leadership style, governance structure and cultural and social norm).

Thus, it is important to strengthen crisis leadership by building a centralized and coordinated public health crisis governance structure and by selecting political elites with leadership styles conducive to crisis management. Additionally, enhancing social cohesion and fostering a cultural and social norm that facilitates state–society collaboration can improve crisis management and strengthen democratic resilience in the future. In this process, we admit that it is not easy to achieve optimal coordination in crisis management (150). Democracies are required to take into account the complexity of their specific government system and governance arrangements. Each democratic country needs to consider its own history and social structure (e.g., the complex federal system in the US), and adopt solutions suitable for itself to improve its ability to respond effectively to crises and enhance the resilience of democracy, so as to be well prepared for future crises. For instance, the US has a more complex federal system than many other Western democracies, which makes it more difficult for the federal government to strengthen its crisis leadership in the US.

Finally, we acknowledge some limitations of this article. First, our analytical framework mainly focuses on the individual, institutional and cultural dimensions of contextual factors, without considering the impact of other contextual factors on public health crisis management. Scholars can expand this analytical framework and incorporate other dimensions (e.g., technology and economic conditions) of contextual factors into the analysis, further strengthening our understanding of how contextual factors affect public health crisis management in democracies in the future. For example, stronger medical technology can provide better medical services for patients, and leading digital technology can promote effective public health crisis management by timely monitoring the spread of viruses and the whereabouts of patients. Countries with good economic conditions are more capable of strengthening investment in public health and healthcare systems, and have more sufficient resources such as medical supplies to respond to public health crises.

Second, due to the epidemic and travel restrictions, the authors did not conduct extensive fieldwork in the US and South Korea during the pandemic period. We acknowledge that the absence of extensive fieldwork in the US and South Korea due to pandemic-related travel restrictions could limit the depth of insights. In the post-COVID-19 era, with the lifting of travel restrictions, surveys and in-depth interviews can be conducted in these two countries to further validate research findings, increasing the depth of insights and data reliability. In the future, it is also possible to collaborate with scholars from democratic countries such as the United States and South Korea through transnational cooperation to conduct relevant research and collect more on-the-ground data, thereby increasing the reliability of this study. In addition to the US and South Korea, future research can examine and compare crisis responses in other democracies to promote more in-depth comparative case studies to validate our findings. The applicability of the framework to other democracies should be explored cautiously.

## Data availability statement

The original contributions presented in the study are included in the article/supplementary material, further inquiries can be directed to the corresponding author.

## Author contributions

YM: Writing – original draft, Writing – review & editing.

## References

[1] Baumgartner FR Carammia M Epp DA Noble B Rey B Yildirim TM. Budgetary change in authoritarian and democratic regimes. J Eur Publ Policy. (2017) 24:792–808. doi: 10.1080/13501763.2017.1296482

[2] Mao Y. Political institutions, state capacity, and crisis management: a comparison of China and South Korea. Int Polit Sci Rev. (2021) 42:316–32. doi: 10.1177/0192512121994026

[3] Goetz KH Martinsen DS. COVID-19: a dual challenge to European liberal democracy. West Eur Polit. (2021) 44:1003–24. doi: 10.1080/01402382.2021.1930463

[4] Keen D. Does democracy protect? The United Kingdom, the United States, and Covid COVI. Disasters. (2021) 45:S26–47. doi: 10.1111/disa.12527 34874072

[5] Engler S Brunner P Loviat R Abou-Chadi T Leemann L Glaser A. Democracy in times of the pandemic: explaining the variation of COVID-19 policies across European democracies. West Eur Polit. (2021) 44:1077–102. doi: 10.1080/01402382.2021.1900669

[6] Greer SL King EJ Fonseca EM Peralta-Santos A. Coronavirus politics: The comparative politics and policy of COVID-19. Ann Arbor, MI: University of Michigan Press (2021).

[7] Steytler N. Comparative federalism and COVID-19: combating the pandemic. London: Routledge (2022).

[8] Parker CF Stern EK. The trump administration and the COVID-19 crisis: exploring the warning-response problems and missed opportunities of a public health emergency. Public Adm. (2022) 100:616–32. doi: 10.1111/padm.12843, PMID: 35601345 PMC9115435

[9] Holloway J Manwaring R. How well does ‘resilience’ apply to democracy? A systematic review. Contemp Polit. (2022) 29:68–92. doi: 10.1080/13569775.2022.2069312

[10] An BY Tang S-Y. Lessons from COVID-19 responses in East Asia: institutional infrastructure and enduring policy instruments. Am. Rev. Public Admin. (2020) 50:790–800. doi: 10.1177/0275074020943707

[11] Capano G Howlett M Jarvis DSL Ramesh M Goyal N. Mobilizing policy (in)capacity to fight COVID-19: understanding variations in state responses. Polic Soc. (2020) 39:285–308. doi: 10.1080/14494035.2020.1787628, PMID: 35039722 PMC8754710

[12] Kavanagh MM Singh R. Democracy, capacity, and coercion in pandemic response: COVID-19 in comparative political perspective. J Health Polit Policy Law. (2020) 45:997–1012. doi: 10.1215/03616878-8641530, PMID: 32464665

[13] Toshkov D Carroll B Yesilkagit K. Government capacity, societal trust or party preferences: what accounts for the variety of national policy responses to the COVID-19 pandemic in Europe? J Eur Publ Policy. (2022) 29:1009–28. doi: 10.1080/13501763.2021.1928270

[14] Yan B Zhang X Wu L Zhu H Chen B. Why do countries respond differently to COVID-19? A comparative study of Sweden, China, France, and Japan. Am Rev Public Admin. (2020) 50:762–9. doi: 10.1177/0275074020942445

[15] Yen WT Liu LY Won E. The imperative of state capacity in public health crisis: Asia's early COVID-19 policy responses. Int J Policy Admin Institut. (2022) 35:777–98. doi: 10.1111/gove.12695 PMC9111679 35601355

[16] Downey DC Myers WM. Federalism, intergovernmental relationships, and emergency response: a comparison of Australia and the United States. Am Rev Public Admin. (2020) 50:526–35. doi: 10.1177/0275074020941696

[17] Bromfield N McConnell A. Two routes to precarious success: Australia, New Zealand, COVID-19 and the politics of crisis governance. Int Rev Adm Sci. (2021) 87:518–35. doi: 10.1177/0020852320972465

[18] Sebhatu A Wennberg K Arora-Jonsson S Lindberg SI. Explaining the homogeneous diffusion of COVID-19 nonpharmaceutical interventions across heterogeneous countries. Proc Acad Natl Sci Phila. (2020) 117:21201–8. doi: 10.1073/pnas.2010625117, PMID: 32788356 PMC7474611

[19] Bollyky TJ Angelino O Wigley S Dieleman JL. Trust made the difference for democracies in COVID-19. Lancet. (2022) 400:657. doi: 10.1016/S0140-6736(22)01532-X, PMID: 36030809 PMC9411258

[20] Comfort LK. Cognition, collective action, and COVID-19: managing crises in real time. Public Perform Manag Rev. (2022) 45:894–915. doi: 10.1080/15309576.2022.2036204

[21] Comfort LK Kapucu N Ko K Menoni S Siciliano M. (2020) Crisis decision‐making on a global scale: transition from cognition to collective action under threat of COVID‐19. Public Adm Rev. 80, 616–622, doi: 10.1111/puar.13252 PMC7300963 32836462

[22] Easton M De Paepe J Evans PW Head B Yarnold J. Embedding expertise for policy responses to COVID-19: comparing decision-making structures in two Federal Democracies. Public Organ Rev. (2022) 22:309–26. doi: 10.1007/s11115-022-00629-6

[23] Parrado S Galli D. Intergovernmental veto points in crisis management: Italy and Spain facing the COVID-19 pandemic. Int Rev Adm Sci. (2021) 87:576–92. doi: 10.1177/0020852320985925

[24] Bejan R Nikolova K. COVID-19 amongst western democracies: a welfare state analysis. Soc Theory Health. (2022) 20:123–51. doi: 10.1057/s41285-022-00178-4, PMID: 35492957 PMC9039269

[25] Croissant A Hellmann O. Democracy, state capacity and the governance of COVID-19 in Asia-Oceania. 1st ed. New York: Routledge (2023).

[26] Institute of Medicine (US) Committee on Assuring the Health of the Public in the 21st Century. The future of the Public’s health in the 21st century. Washington DC: National Academies Press (US) (2003).

[27] Kettl DF. States divided: the implications of American federalism for COVIDD-19. Public Adm Rev. (2020) 80:595–602. doi: 10.1111/puar.13243, PMID: 32836439 PMC7280573

[28] Birkland TA Taylor K Crow DA DeLeo R. Governing in a polarized era: federalism and the response of U.S. state and Federal Governments to the COVID-19 pandemic. Publius. (2021) 51:650–72. doi: 10.1093/publius/pjab024

[29] Foreman CH. Plagues, products, and politics: Emergent public health hazards and national policymaking. Washington DC: Brookings Institution Press (1994).

[30] Garrett L (2000) Betrayal of trust: The collapse of global public health (1st ed). New York: Hyperion, 1, 461–462

[31] Boin A Hart P Stern E. The politics of crisis management: public leadership under pressure. 2nd ed. Cambridge: Cambridge University Press (2017).

[32] Wu YL Shao B Newman A Schwarz G. Crisis leadership: a review and future research agenda. Leadersh Q. (2021) 32:101518. doi: 10.1016/j.leaqua.2021.101518

[33] Kapucu N Ustun Y. Collaborative crisis management and leadership in the public sector. Int J Public Adm. (2018) 41:548–61. doi: 10.1080/01900692.2017.1280819

[34] McGuire M Silvia C. Does leadership in networks matter? Examining the effect of leadership behaviors on managers’ perceptions of network effectiveness. Public Perform Manag Rev. (2009) 33:34–62. doi: 10.2753/PMR1530-9576330102

[35] Waugh WL Jr Streib G. Collaboration and leadership for effective emergency management. Public Adm Rev. (2006) 66:131–40. doi: 10.1111/j.1540-6210.2006.00673.x

[36] Yang K. Unprecedented challenges, familiar paradoxes: COVID-19 and governance in a new normal state of risks. Public Adm Rev. (2020) 80:657–64. doi: 10.1111/puar.13248, PMID: 32836451 PMC7283883

[37] Song M Park HJ Jung K. Do political similarities facilitate interlocal collaboration? Public Adm Rev. (2018) 78:261–9. doi: 10.1111/puar.12887

[38] Mao Y. Combating COVID-19 through collaborative governance: lessons from East Asia. Chin Public Admin Rev. (2020) 11:132–41. doi: 10.22140/cpar.v11i2.255

[39] Zhao T Wu ZS. Citizen–state collaboration in combating COVID-19 in China: experiences and lessons from the perspective of co-production. Am Rev Public Admin. (2020) 50:777–83. doi: 10.1177/0275074020942455

[40] Carayannopoulos G. Whole of government: the solution to managing crises? Aust J Public Adm. (2017) 76:251–65. doi: 10.1111/1467-8500.12227

[41] Weible CM Nohrstedt D Cairney P Carter DP Crow DA Durnová AP. COVID-19 and the policy sciences: initial reactions and perspectives. Policy Sci. (2020) 53:225–41. doi: 10.1007/s11077-020-09381-4, PMID: 32313308 PMC7165254

[42] Petridou E Zahariadis N. Staying at home or going out? Leadership response to the COVID facing the COVID-19 and Sweden. J Contingencies Crisis Manage. (2021) 29:293–302. doi: 10.1111/1468-5973.12344

[43] Mao Y. What accounts for the different regional responses to COVID-19 in China? Exploring the role of institutional environment, governance capacity and legitimacy. Health Policy Plan. (2023) 38:552–66. doi: 10.1093/heapol/czad007, PMID: 36715072

[44] Christensen T Lægreid P Rykkja LH. Organizing for crisis management: building governance capacity and legitimacy. Public Adm Rev. (2016) 76:887–97. doi: 10.1111/puar.12558

[45] Lodge M Wegrich K eds. The problem-solving capacity of the modern state: governance challenges and administrative capacities. Oxford: Oxford University Press (2014).

[46] Boin A McConnell A Hart P. Governing the pandemic: The politics of navigating a mega-crisis Palgrave Macmillan (2021). Available at: https://link.springer.com/book/10.1007/978-3-030-72680-5

[47] Howlett M. Policy analytical capacity: the supply and demand for policy analysis in government. Polic Soc. (2015) 34:173–82. doi: 10.1016/j.polsoc.2015.09.002

[48] Comfort LK. Crisis management in hindsight: cognition, communication, coordination, and control. Public Adm Rev. (2007) 67:189–97. doi: 10.1111/j.1540-6210.2007.00827.x

[49] Rosenthal U Kouzmin A. Crises and crisis management: toward comprehensive government decision making. J Public Adm Res Theory. (1997) 7:277–304. doi: 10.1093/oxfordjournals.jpart.a024349

[50] Van Wart M Kapucu N. Crisis management competencies: the case of emergency managers in the USA. Public Manag Rev. (2011) 13:489–511. doi: 10.1080/14719037.2010.525034

[51] Van Bavel JJ Baicker K Boggio PS Capraro V Cichocka A Cikara M. Using social and behavioural science to support COVID-19 pandemic response. Nat Hum Behav. (2020) 4:460–71. doi: 10.1038/s41562-020-0884-z 32355299

[52] Jae Moon M Suzuki K Park TI Sakuwa K. A comparative study of COVID-19 responses in South Korea and Japan: political nexus triad and policy responses. Int Rev Adm Sci. (2021) 87:651–71. doi: 10.1177/0020852321997552 PMC8685564 40479109

[53] Farazmand A. Learning from the Katrina crisis: a global and international perspective with implications for future crisis management. Public Adm Rev. (2007) 67:149–59. doi: 10.1111/j.1540-6210.2007.00824.x

[54] Boin A Hart P‘T McConnell A Preston T. Leadership style, crisis response and blame management: the case of hurricane Katrina. Public Adm. (2010) 88:706–23. doi: 10.1111/j.1467-9299.2010.01836.x

[55] Dasborough MT Scandura T. Leading through the crisis: “hands off” or “hands-on”? J Leader Organ Stud. (2022) 29:219–23. doi: 10.1177/15480518211036472

[56] Dyson SB Preston T. Individual characteristics of political leaders and the use of analogy in foreign policy decision making. Polit Psychol. (2006) 27:265–88. doi: 10.1111/j.1467-9221.2006.00006.x

[57] Preston T Hart P ‘t (1999) Understanding and evaluating bureaucratic politics: the nexus between political leaders and advisory systems. Polit Psychol 20, 49–98, doi: 10.1111/0162-895X.00137

[58] Green J Edgerton J Naftel D Shoub K Cranmer SJ. Elusive consensus: polarization in elite communication on the COVID-19 pandemic. Science. Advances. (2020) 6:eabc2717. doi: 10.1126/sciadv.abc2717 PMC7455486 32923600

[59] Falkenbach M Willison C. Resources or trust: what matters more in the vaccination strategies of high-income liberal democracies? Health Policy Technol. (2022) 11:100618. doi: 10.1016/j.hlpt.2022.100618, PMID: 35369129 PMC8956345

[60] Flores A Cole JC Dickert S Eom K Jiga-Boy GM Kogut T. Politicians polarize and experts depolarize public support for COVID-19 management policies across countries. Proceed Natl Acad Sci. (2022) 119:e2117543119. doi: 10.1073/pnas.2117543119, PMID: 35042779 PMC8784107

[61] Ramírez de la Cruz EE Grin EJ SanabriaPulido P Cravacuore D Orellana A. The Transaction Costs of Government Responses to the COVID19 Emergency in Latin America. Public Admini. Rev.. (2020) 80:683–695. 10.1111/puar.13259 PMC7300791 32836458

[62] Kerr J Panagopoulos C Van der Linden S. Political polarization on COVID-19 pandemic response in the United States. Personal Individ Differ. (2021) 179:110892. doi: 10.1016/j.paid.2021.110892, PMID: 34866723 PMC8631569

[63] Iyengar S Lelkes Y Levendusky M Malhotra N Westwood SJ. The origins and consequences of affective polarization in the United States. Annu Rev Polit Sci. (2019) 22:129–46. doi: 10.1146/annurev-polisci-051117-073034

[64] Druckman JN Klar S Krupnikov Y Levendusky M Ryan JB. How affective polarization shapes Americans’ political beliefs: a study of response to the COVID-19 pandemic. J Experiment Polit Sci. (2021) 8:223–34. doi: 10.1017/XPS.2020.28

[65] Merkley E Bridgman A Loewen PJ Owen T Ruths D Zhilin O. A rare moment of cross-partisan consensus: elite and public response to the COVID-19 pandemic in Canada. Can J Polit Sci. (2020) 53:311–8. doi: 10.1017/S0008423920000311

[66] Borgonovi F Andrieu E Subramanian SV. The evolution of the association between community level social capital and COVID-19 deaths and hospitalizations in the United States. Soc Sci Med. (2021) 278:113948. doi: 10.1016/j.socscimed.2021.113948, PMID: 33930677 PMC8055504

[67] Makridis CA Wu C. How social capital helps communities weather the COVID-19 pandemic. PLoS One. (2021) 16:e0245135. doi: 10.1371/journal.pone.0245135, PMID: 33513146 PMC7846018

[68] Bynander F Nohrstedt D eds. Collaborative crisis management: inter-organizational approaches to extreme events. New York: Routledge (2020).

[69] Moon MJ. Fighting COVID for all - president Moon Jae-in’s speech for the 73rd world health assembly. PublicAdmin Rev. (2020) 80:651–6. doi: 10.1111/puar.13214

[70] Tsai LL Morse BS Blair RA. Building credibility and cooperation in low-trust settings: persuasion and source accountability in Liberia during the 2014–2015 Ebola crisis. Comp Pol Stud. (2020) 53:1582–618. doi: 10.1177/0010414019897698

[71] Gerring J. Case study research: Principles and practices. New York: Cambridge University Press (2007).

[72] Johns Hopkins Center for Health Security, Nuclear Threat Initiative and Economist Intelligence Unit (2019) 2019 Global Health security index report. Available at: https://www.ghsindex.org/wp-content/uploads/2019/10/2019-Global-Health-Security-Index.pdf

[73] Lippman D Mcgraw M (2020) Inside the National Security Council, a rising sense of dread. Politico. Available at: https://www.politico.com/news/2020/04/02/nsc-coronavirus-white-house-162530

[74] Litpon E Sanger D Haberman M Shear MD Mazzetti M Barnes JE. (2020) He could have seen what was coming: Behind Trump’s failure on the virus. The New York Times. Available at: https://www.nytimes.com/2020/04/11/us/politics/coronavirus-trump-response.html

[75] Cohen Z Sciutto J Marquardt A Perez E. (2020), US intelligence agencies started tracking coronavirus outbreak in China as early as November. CNN. Available at: https://edition.cnn.com/2020/04/08/politics/intel-agencies-covid-november/index.html

[76] CNBC (2020) CNBC transcript: President Donald Trump sits down with CNBC’s joe Kernen at the world economic forum in Davos, Switzerland: CNBC. Available at: https://www.cnbc.com/2020/01/22/cnbc-transcript-president-donald-trump-sits-down-with-cnbcs-joe-kernen-at-the-world-economic-forum-in-davos-switzerland.html

[77] Leonhardt D (2020) A complete list of Trump’s attempts to play down coronavirus. The New York Times. Available at: https://www.nytimes.com/2020/03/15/opinion/trump-coronavirus.html

[78] WHO (2020), Mission summary: WHO field visit to Wuhan, China 20-21 January 2020. Available at: https://www.who.int/china/news/detail/22-01-2020-field-visit-wuhan-china-jan-2020

[79] Diamond D Tossi N (2020, March 25) Trump team failed to follow NSCs pandemic playbook. Politico. Available at: https://www.politico.com/news/2020/03/25/trump-coronavirus-national-security-council-149285

[80] U.S. Department of Health and Human Services (2020) Determination that a public health emergency exists. Available at: https://www.phe.gov/emergency/news/healthactions/phe/Pages/2019-nCoV.aspx

[81] Moon S Yan H Christensen J Maxouris C. (2020), The CDC has changed its criteria for testing patients for coronavirus after the first case of unknown origin was confirmed. CNN. Available at: https://edition.cnn.com/2020/02/27/health/us-cases-coronavirus-community-transmission/index.html

[82] Shear MD Weiland N Rogers K (2020) Trump names Mike pence to Lead coronavirus response. The New York Times. Available at: https://www.nytimes.com/2020/02/26/us/politics/trump-coronavirus-cdc.html

[83] White House (2020) Proclamation on declaring a National Emergency Concerning the novel coronavirus disease (COVID-19) outbreak. Available at: https://trumpwhitehouse.archives.gov/presidential-actions/proclamation-declaring-national-emergency-concerning-novel-coronavirus-disease-covid-19-outbreak/

[84] White House (2020) 15 days to slow the spread. Available at: https://trumpwhitehouse.archives.gov/articles/15-days-slow-spread/

[85] Biesecker M Stobbe M and Perrone (2020) Testing blunders crippled US response as coronavirus spread. AP News. Available at: https://apnews.com/article/public-health-united-nations-donald-trump-ap-top-news-virus-outbreak-c335958b1f8f6a37b19b421bc7759722

[86] Ballhaus R Armour S. (2020). Health chief’s missteps set back virus response—HHS secretary Azar waited weeks to brief Trump, oversold progress. Wall Street Journal. Available at: https://www.wsj.com/articles/health-chiefs-early-missteps-set-back-coronavirus-response-11587570514

[87] Koronowski R Venook J Ragland W (2020) ‘Blinking red’: A running timeline of how the trump administration ignored warnings, misled the public, and made the coronavirus crisis worse. The Center for American Progress. Available at: https://www.americanprogress.org/article/blinking-red-trump-administration-ignored-warnings-misled-public-made-coronavirus-crisis-worse/

[88] World Bank (2023). World Bank Open Data. Available at: https://data.worldbank.org

[89] Korea Statistical Information Service (2023). Projected population by age group (Korea). Available at: https://kosis.kr/statHtml/statHtml.do?orgId=101&tblId=DT_1IN1502&vw_cd=MT_ETITLE&list_id=A11_2015_1&scrId=&language=en&seqNo=&lang_mode=en&obj_var_id=&itm_id=&conn_path=MT_ETITLE&path=%252Feng%252FstatisticsList%252FstatisticsListIndex.do

[90] Census Bureau U.S. (2022). American community survey 1-year estimates. Retrieved from Census Reporter Profile page for United States. Available at: http://censusreporter.org/profiles/01000US-united-states/

[91] Lim S Sohn M. How to cope with emerging viral diseases: lessons from South Korea’s strategy for COVID-19, and collateral damage to cardiometabolic health. Lancet Regional Health Western Pacific. (2023) 30:100581. doi: 10.1016/j.lanwpc.2022.100581, PMID: 36093123 PMC9442269

[92] WHO (2023) WHO COVID-19 database. Available at: https://covid19.who.int

[93] Bloomberg (2022) The Bloomberg’s Covid resilience ranking. Available at: https://www.bloomberg.com/graphics/covid-resilience-ranking/

[94] Ang YY. When COVID-19 meets centralized, personalized power. Nat Hum Behav. (2020) 4:445–7. doi: 10.1038/s41562-020-0872-3, PMID: 32273583

[95] Gakh M Ha J Won Yoo J Han DH. Preparing for the next pandemic: learning lessons from the Republic of Korea to bolster public health disease surveillance in the United States. Health Security. (2022) 20:177–81. doi: 10.1089/hs.2021.0151, PMID: 35319262

[96] Issac A Stephen S Jacob J VR V Radhakrishnan RV Krishnan N. The pandemic league of COVID-19: Korea versus the United States, with lessons for the entire world. J Prev Med Public Health. (2020) 53:228–32. doi: 10.3961/jpmph.20.166, PMID: 32752591 PMC7411243

[97] Lee S Yeo J Na C. Learning before and during the COVID-19 outbreak: a comparative analysis of crisis learning in South Korea and the US. Int Rev Public Admin. (2020) 25:243–60. doi: 10.1080/12294659.2020.1852715

[98] Park S Maher CS. Government financial management and the coronavirus pandemic: a comparative look at South Korea and the United States. Am Rev Public Admin. (2020) 50:590–7. doi: 10.1177/0275074020941720

[99] Kapucu N Moynihan D. Trump’s (mis)management of the COVID-19 pandemic in the US. Policy Studies. (2021) 42:592–610. doi: 10.1080/01442872.2021.1931671

[100] The Centers for Disease Control and Prevention (2021) CDC’s response. Available at: https://www.cdc.gov/coronavirus/2019-ncov/cdcresponse/index.html

[101] Glenn J Chaumont C Villalobos Dintrans P. Public health leadership in the times of COVID-19: a comparative case study of three countries. Int J Public Leader. (2021) 17:81–94. doi: 10.1108/IJPL-08-2020-0082

[102] Siciliano MD Comfort LK Kapucu N Lee S Li J. Same country, different stories: context, complexity, and cognition in the United States In: Comfort LK Rhodes ML, editors. Global risk management: The role of collective cognition in response to COVID-19. New York, NY: Routledge (2022).

[103] Kuznia R Devine C Valencia N (2020) ‘We’ve been muzzled’: CDC sources say white house putting politics ahead of science. CNN. Available at: https://www.cnn.com/2020/05/20/politics/coronavirus-travel-alert-cdc-white-house-tensions-invs/index.html

[104] McLaughlin EC Almasy S (2020) CDC official warns Americans it’s not a question of if coronavirus will spread, but when. CNN. Available at: https://edition.cnn.com/2020/02/25/health/coronavirus-us-american-cases/index.html

[105] Diamond D Tossi N (2020) Trump team failed to follow NSC’s pandemic playbook. Available at: Politico. https://www.politico.com/news/2020/03/25/trump-coronavirus-national-security-council-149285

[106] Riechmann D (2020) Trump disbanded NSC pandemic unit that experts had praised. Associated Press News. Available at: https://apnews.com/article/donald-trump-ap-top-news-virus-outbreak-barack-obama-public-health-ce014d94b64e98b7203b873e56f80e9a

[107] Cameron B (2020), I ran the white house pandemic office. Trump closed it. The Washington Post. Available at: https://www.washingtonpost.com/outlook/nsc-pandemic-office-trump-closed/2020/03/13/a70de09c-6491-11ea-acca-80c22bbee96f_story.html

[108] Schismenos S Smith AA Stevens GJ Emmanouloudis D. Failure to lead on COVID-19: what went wrong with the United States? Int J Public Leadership. (2021) 17:39–53. doi: 10.1108/IJPL-08-2020-0079

[109] Oh S-Y (2020) South Korea’s success against COVID-19. The regulatory Review. Available at: https://www.theregreview.org/2020/05/14/oh-south-korea-success-against-covid-19/

[110] The Government of the Republic of Korea (2020), All about Korea’s response to COVID-19. Available at: https://www.mofa.go.kr/eng/brd/m_22591/view.do?seq=35&srchFr=&srchTo=&srchWord=&srchTp=&multi_itm_seq=0&itm_seq_1=0&itm_seq_2=0&company_cd=&company_nm=&page=1&titleNm=

[111] Moon J (2020) Remarks by president Moon Jae-in at appointment ceremony for new commissioner of Korea disease control and prevention agency. Available at: https://english1.president.go.kr/BriefingSpeeches/Others/874

[112] Reuters (2020). Timeline - in his own words: Trump and the coronavirus. Available at: https://www.reuters.com/article/us-health-coronavirus-usa-trump-comments-idUSKBN26N0U5

[113] Forgey Q (2020) ‘We’re not a shipping clerk’: Trump tells governors to step up efforts to get medical supplies. Politico. Available at: https://www.politico.com/news/2020/03/19/trump-governors-coronavirus-medical-supplies-137658

[114] Wilkie C Breuninger K (2020) Trump says he told pence not to call governors who aren’t ‘appreciative’ of white house coronavirus efforts. CNBC. Available at: https://www.cnbc.com/2020/03/27/coronavirus-trump-told-pence-not-to-call-washington-michigan-governors.html

[115] Abutaleb Y McGinley L (2020) Ousted vaccine official alleges he was demoted for prioritizing ‘science and safety’. The Washington Post. Available at: https://www.washingtonpost.com/health/2020/05/05/rick-bright-hydroxychloroquine-whistleblower-complaint/

[116] CDC COVID-19 Response Team (2020) Characteristics of health care personnel with COVID-19 – United States, February 12–April 9, 2020. Available at: https://www.cdc.gov/mmwr/volumes/69/wr/mm6915e6.htm 10.15585/mmwr.mm6915e6 PMC7755055 32298247

[117] Dawsey J Parker A Rucker P Abutaleb Y. (2020) As deaths mount, trump tries to convince Americans it’s safe to inch back to normal. The Washington Post. Available at: https://www.washingtonpost.com/politics/as-deaths-mount-trump-tries-to-convince-americans-its-safe-to-inch-back-to-normal/2020/05/09/bf024fe6-9149-11ea-a9c0-73b93422d691_story.html

[118] Select subcommittee on the coronavirus crisis (2022) “It was compromised”: the Trump Administration’s unprecedented campaign to control CDC and politicize public health during the coronavirus crisis: staff report.

[119] Piller C. Undermining CDC: Deborah Birx, president Donald Trump’s COVID-19 coordinator, helped shake the foundation of a premier public health agency. Science. (2020) 370:394–9. doi: 10.1126/science.370.6515.394 33093092

[120] Hahm SD Heo U. President Moon Jae-in at midterm: what affects public support for Moon Jae-in? J Asian Afr Stud. (2020) 55:1128–42. doi: 10.1177/0021909620911145

[121] Ministry of Foreign Affairs, Republic of Korea (2020) Korea’s evolving response to COVID-19. Available at: https://overseas.mofa.go.kr/my-en/brd/m_21422/view.do?seq=53&srchFr=&srchTo=&srchWord=&srchTp=&multi_itm_seq=0&itm_seq_1=0&itm_seq_2=0&company_cd=&company_nm=&page=6

[122] You J. Lessons from South Korea’s COVID-19 policy response. Am Rev Public Admin. (2020) 50:801–8. doi: 10.1177/0275074020943708

[123] Xu HD Basu R. How the United States flunked the COVID-19 test: some observations and several lessons. Am Rev Public Admin. (2020) 50:568–76. doi: 10.1177/0275074020941701

[124] Arit J (2020) These eight states haven’t issued stay-at-home orders to fight the coronavirus outbreak. Los Angeles Times. Available at: https://www.latimes.com/politics/story/2020-04-22/states-without-coronavirus-stay-at-home-order

[125] Moradi H Vaezi A. Lessons learned from Korea: COVID-19 pandemic. Infect Control Hospital Epidemiol. (2020) 41:873–4. doi: 10.1017/ice.2020.104, PMID: 32241308 PMC7167487

[126] Moon J (2020). Remarks by president Moon Jae-in at G20 2020 extraordinary virtual leaders summit. Available at: https://english1.president.go.kr/Briefingspeeches/Speeches/786

[127] Kim A (2020) Immediate action needed to avoid health system collapse: KCDC chief warns of virus risk lurking in ‘everyday places’ around Seoul. The Korea Herald. Available at: https://www.koreaherald.com/view.php?ud=20200817000037

[128] Gabby O Levine M (2020) Trump’s #FireFauci retweet spurs a cycle of outrage and a white house denial. Politico. Available at: https://www.politico.com/news/2020/04/13/trump-fauci-fire-tweet-coronavirus-183907

[129] Charoenwong B Kwan A Pursiainen V. Social connections with COVID-19-affected areas increase compliance with mobility restrictions. Science. Advances. (2020) 6:eabc3054. doi: 10.1126/sciadv.abc3054 PMC10662649 33097473

[130] Shin G-W. South Korea’s democratic decay. J Democr. (2020) 31:100–14. doi: 10.1353/jod.2020.0048

[131] Sang-Hun C (2020) In South Korea’s new COVID-19 outbreak, religion and politics collide. The New York Times. Available at: https://www.nytimes.com/2020/08/20/world/asia/coronavirus-south-korea-church-sarang-jeil.html

[132] Rich TS Eliassen I Dahmer A Einhorn M Woggon E Bison K. (2020), South Korea: the politics of COVID-19. The Diplomat. Available at: https://thediplomat.com/2020/03/south-korea-the-politics-of-covid-19/

[133] Kumar R. Bringing the developmental state back in: explaining South Korea’s successful management of COVID-19. Third World Q. (2021) 42:1397–416. doi: 10.1080/01436597.2021.1903311

[134] Hyonhee S (2020) South Korea’s coronavirus battle propels Moon’s party to election win. Reuters. Available at: https://www.reuters.com/article/us-health-coronavirus-southkorea-electio/south-koreas-coronavirus-battle-propels-moons-party-to-election-win-idUSKCN21Y097

[135] Gelfand MJ Nishii LH Raver JL. On the nature and importance of cultural tightness-looseness. J Appl Psychol. (2006) 91:1225–44. doi: 10.1037/0021-9010.91.6.1225, PMID: 17100480

[136] Gelfand MJ. Rule makers, rule breakers: how tight and loose cultures wire our world. New York: Scribner (2018).

[137] Gelfand MJ Raver JL Nishii LH Leslie LM Lun J Lim BC. Differences between tight and loose cultures: a 33-nation study. Science. (2011) 332:1100–4. doi: 10.1126/science.1197754, PMID: 21617077

[138] Gelfand MJ. Culture’s constraints: international differences in the strength of social norms. Curr Dir Psychol Sci. (2012) 21:420–4. doi: 10.1177/0963721412460048

[139] Gelfand MJ Harrington JR Jackson JC. The strength of social norms across human groups. Perspect Psychol Sci. (2017) 12:800–9. doi: 10.1177/1745691617708631 28972845

[140] Bazzi S Fiszbein M Gebresilasse M. “Rugged individualism” and collective (in)action during the COVID-19 pandemic. J Public Econ. (2021) 195:104357. doi: 10.1016/j.jpubeco.2020.104357

[141] Gelfand MJ Jackson JC Pan X Nau D Pieper D Denison E. The relationship between cultural tightness–looseness and COVID-19 cases and deaths: a global analysis. Lancet Planetary Health. (2021) 5:e135–44. doi: 10.1016/S2542-5196(20)30301-6, PMID: 33524310 PMC7946418

[142] Putnam RD. Bowling alone: Revised and updated: The collapse and revival of American community. New York: Simon and Schuster (2020).

[143] News BBC (2020) Coronavirus lockdown protest: what’s behind the US demonstrations? Available at: https://www.bbc.com/news/world-us-canada-52359100

[144] Harrington JR Gelfand MJ. Tightness–looseness across the 50 United States. Proceed Natl Acad Sci. (2014) 111:7990–5. doi: 10.1073/pnas.1317937111, PMID: 24843116 PMC4050535

[145] Erkoreka M Hernando-Pérez J. Decentralization: a handicap in fighting the COVID-19 pandemic? The response of the regional governments in Spain. Public Adm Dev. (2022) 43:129–40. doi: 10.1002/pad.1988 PMC9349406 35942434

[146] Moon J (2020) Freedom for all - president Moon Jae-in’s speech for the 73rd world health assembly. Available at: https://overseas.mofa.go.kr/us-houston-en/brd/m_5573/view.do?seq=759772

[147] Lee D Heo K Seo Y. COVID-19 in South Korea: lessons for developing countries. World Dev. (2020) 135:105057. doi: 10.1016/j.worlddev.2020.105057, PMID: 32834374 PMC7321039

[148] Yin RK. Validity and generalization in future case study evaluations. Evaluation. (2013) 19:321–32. doi: 10.1177/1356389013497081

[149] Bauer MW Peters BG Pierre J Yesilkagit K Becker S. Democratic backsliding and public administration: how populists in government transform state bureaucracies. Cambridge: Cambridge University Press (2021).

[150] Farrar J Ahuja A. Spike: the virus vs. the people – the inside story Profile Books (2021).

